# Prediction of ACE-I Inhibitory Peptides Derived from Chickpea (*Cicer arietinum* L.): In Silico Assessments Using Simulated Enzymatic Hydrolysis, Molecular Docking and ADMET Evaluation

**DOI:** 10.3390/foods11111576

**Published:** 2022-05-27

**Authors:** Jesús Gilberto Arámburo-Gálvez, Aldo Alejandro Arvizu-Flores, Feliznando Isidro Cárdenas-Torres, Francisco Cabrera-Chávez, Giovanni I. Ramírez-Torres, Lilian Karem Flores-Mendoza, Pedro Erick Gastelum-Acosta, Oscar Gerardo Figueroa-Salcido, Noé Ontiveros

**Affiliations:** 1 Postgraduate in Health Sciences, Division of Biological and Health Sciences, University of Sonora, Hermosillo 83000, Mexico; gilberto.aramburo@uas.edu.mx (J.G.A.-G.); aldo.arvizu@unison.mx (A.A.A.-F.); 2 Nutrition Sciences Postgraduate Program, Faculty of Nutrition Sciences, University of Sinaloa, Culiacan 80019, Mexico; feliznando@uas.edu.mx (F.I.C.-T.); fcabrera@uas.edu.mx (F.C.-C.); giovanni.ramirez@uas.edu.mx (G.I.R.-T.); 3 Faculty of Physical Education and Sports, University of Sinaloa, Culiacan 80013, Mexico; erickgastelum@uas.edu.mx; 4 Clinical and Research Laboratory (LACIUS, URS), Department of Chemical, Biological, and Agricultural Sciences (DC-QB), Division of Sciences and Engineering, University of Sonora, Navojoa 85880, Mexico; lilian.flores@unison.mx

**Keywords:** ACE-I, antihypertensive peptides, hypertension, bioactive peptides, chickpea, molecular docking, in silico, BIOPEP

## Abstract

Chickpea (*Cicer arietinum* L.) peptides have shown in vitro potential to inhibit the angiotensin I-converting enzyme (ACE-I). However, the potential molecular interactions between chickpea peptides (CP) and ACE-I as well as their ADMET (absorption/distribution/metabolism/excretion/toxicity) characteristics remain unknown. Thus, our aim was to study the in silico interactions of CP with ACE-I and the CP ADMET characteristics. Legumin and provicilin sequences were submitted to in silico analysis to search for ACE-I inhibitory peptides. Simulated enzymatic hydrolysis was performed using the BIOPEP-UWM database, and the ACE-I inhibitory peptides generated (EC50 ≤ 200 μM) were selected to perform molecular docking and ADMET analysis. After hydrolysis, 59 out of 381 peptides with ACE-I inhibitory potential were released. Based on A and B parameters, the legumin peptides showed better ACE-I inhibitory potential than the provicilin ones. CP mainly interact with residues from pocket S1 (Ala354/Glu384) and S2 (His353/His513) through hydrogen bonds (distances < 3.0 Å) and hydrophobic interactions (binding energy from −5.7 to −9.2 kcal/mol). Through ADMET analysis, CP showed optimal values for inhibiting ACE-I in vivo. ACE-I inhibitory peptides from legumin and provicilin can bind strongly and tightly to the active site of ACE-I. Further studies to evaluate in vivo the antihypertensive effects of CP are warranted.

## 1. Introduction

Hypertension is defined as systolic or diastolic blood pressure ≥ 130 or ≥ 80 mm Hg (stage 1), respectively, or systolic or diastolic blood pressure ≥140 or ≥ 90 mm Hg (stage 2), respectively [1], and it is also one of the main risk factors for developing cardiovascular diseases such as heart failure, stroke, and atherosclerosis [2]. Hypertension management involves lifestyle changes (e.g., diet changes and physical activity) and the use of blood pressure-lowering drugs such as diuretics, calcium channel agonists, angiotensin II receptor blockers, and angiotensin I-converting enzyme (ACE; peptidyldipeptide hydrolase, EC 3.4.15.1) inhibitors [3]. ACE-I is a zinc metallopeptidase that hydrolyzes the inactive decapeptide angiotensin I, giving rise to the potent vasoconstrictor octapeptide angiotensin II. Furthermore, ACE-I cleaves the vasodilator bradykinin, further increasing blood pressure. Thus, ACE-I is a therapeutic target for lowering blood pressure. In fact, synthetic ACE-I competitive inhibitors, such as lisinopril, captopril, and enalapril, are commonly used for lowering blood pressure, although they can trigger adverse reactions such as cough, skin rashes, and angioedema [4]. Therefore, natural side-effect-free ACE-I inhibitors for the prevention and management of hypertension are desirable [4].

Chickpea (*Cicer arietinum* L.) is the third most important pulse in the world based on total grain production, and it contains bioactive compounds with antidiabetic, antioxidant, antihypertensive, anti-inflammatory, and anticarcinogenic activity [5]. In silico and in vitro studies have highlighted that chickpea hydrolysates can inhibit ACE-I [6,7,8]. However, chickpea peptides (CP) with ACE-I inhibitory potential are poorly characterized, and there is a lack of studies that evaluate their effects in vivo or their molecular interactions with ACE-I. Knowledge about the potential molecular interactions between CP and ACE-I as well as about the peptides’ predicted pharmacokinetic properties can help us to understand the potential in vivo antihypertensive effects of these peptides. Therefore, this study assesses the CP interactions with active sites of ACE-I using molecular docking and evaluates the potential pharmacokinetic properties of ACE-I inhibitory peptides from chickpea proteins using ADMET analysis.

## 2. Materials and Methods

### 2.1. Protein Sequences 

**Figure 1:**
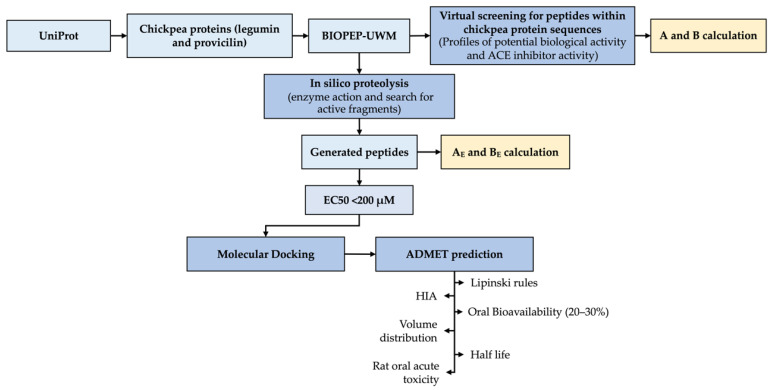
Workflow employed to characterize CP with ACE-I inhibitory capacity. A: frequency of ACE-I-inhibitory fragments, B: potential ACE-I inhibitory activity, A_E_: frequency of released fragments with ACE-I-inhibitory activity, B_E_: ACE-I-inhibitory activity of peptides potentially released by enzymatic hydrolysis, HIA: human intestinal absorption.

The protein sequences of the main storage proteins in chickpea seeds (*Cicer arietinum* L.) were obtained from the UniProt database (http://www.uniprot.org (accessed on 6 December 2021)): legumin (UniProt ID: Q9SMJ4) and provicilin (UniProt ID: Q304D4). The BIOPEP-UWM™ database of bioactive peptides [9] was used to obtain the ACE-I inhibitory peptide predictions from the sequences selected and to carry out in silico enzymatic hydrolysis using pepsin, trypsin, chymotrypsin (A), papain, and alcalase. Afterward, molecular docking and ADMET (absorption, distribution, metabolism, excretion, and toxicity) prediction analyses were performed. A half-maximal effective concentration (EC50) < 200 μM was utilized as a cutoff to carry out molecular docking and ADMET predictions. ACE-I inhibitory peptides without EC50 values available on the BIOPEP-UWM database were not considered for molecular dockings and ADMET analyses. Figure 1 summarizes all the analyses carried out to characterize chickpea ACE-I inhibitory peptides.

### 2.2. Analysis of ACE-I Inhibitory Peptides from Chickpea (Cicer arietinum L.) Proteins

Chickpea seed protein sequences (legumin and provicilin) were evaluated for the prediction of ACE-I inhibitory peptides using the BIOPEP-UWM database [9]. The predicted ACE-I-inhibitory peptides were matched with those in the BIOPEP-UWM database, which contains 1063 peptide sequences with ACE-I-inhibitory activity demonstrated either in vitro or in vivo (consulted in December 2021).

Computer-aided quantification parameters of chickpea proteins were calculated using the tool “Calculation” available in the BIOPEP-UWM database [9]. The following quantification parameters from the legumin and provicilin sequences were determined: (1) the frequency of ACE-I inhibitory fragment occurrence (A) and (2) the potential ACE-I inhibitory activity (B) [µM-1]. Parameter A enables us to determine the frequency of peptide sequences of chickpea proteins with predicted ACE-I inhibitory activity. Higher values of A correspond to more ACE-I inhibitory peptide sequences. Parameter B allows estimation of the potential or “strength” of a protein sequence to have a certain biological activity. Protein/peptide sequences with lower values of B indicate higher ACE-I inhibitory potential. Parameters A and B were calculated as follows [10]:

$$A=\frac{a}{N}$$

a—the number of fragments with ACE-I-inhibitory activity.

N—the number of amino acid residues of the protein.

$$B=\frac{\sum_{i=1}^{k}\frac{a_{i}}{EC_{50i}}}{N}$$

a_i_—the number of repetitions of ACE-I-inhibitory fragments in the protein sequence.

k—the number of different ACE-I-inhibitory fragments.

N—the number of amino acid residues in the protein sequence.

EC_50i_–the half-maximal effective concentration.

### 2.3. In Silico Proteolysis Analysis and Virtual Screening of ACE-I Inhibitory Peptides

Legumin and provicilin sequences were submitted to in silico enzymatic hydrolysis using the “enzymatic action” tool available in the BIOPEP-UWM database [9]. Single hydrolysis analyses were carried out with pepsin (pH 1.3, EC 3.4.23.1), trypsin (EC 3.4.21.4), chymotrypsin (A) (EC 3.4.21.1), papain (EC 3.4.22.2), and alcalase (EC 3.4.21.62). Simultaneous hydrolysis analysis was carried out with pepsin, trypsin, and chymotrypsin to simulate gastrointestinal digestion. The ACE-I inhibitory activity of the fragments obtained was analyzed with the “search for active fragments” tool of the BIOPEP-UWM database.

The following quantification parameters were calculated from the peptides released from the enzymatic hydrolysis: (1) the frequency of peptides released with ACE-inhibitory activity by a given enzyme (A_E_) and (2) the ACE-I inhibitory potential of peptides released by a given enzyme (B_E_). Higher values of A_E_ correspond to more peptides released with ACE-I inhibitory activity. Contrary, lower values of B_E_ equal a higher ACE-I inhibitory potential of peptides released by a given enzyme. Parameters A_E_ and B_E_ were calculated as follows:

$$A_{E}=\frac{d}{N}$$

d—the number of ACE-inhibitory peptides that are released by a given enzyme or enzymes.

N—the number of amino acid residues in the target protein.

$$B_{E}=\frac{\sum_{j=1}^{I}\frac{d_{j}}{EC_{50j}}}{N}$$

d_j_—the number of repeated ACE-I inhibitory peptides that are released by a given enzyme or enzymes from a protein sequence.

I—the number of different ACE-I inhibitory peptides that are released by a given enzyme or enzymes from a protein sequence.

N—the number of amino acid residues in a protein sequence.

EC_50j_—half-maximal effective concentration.

### 2.4. Molecular Docking of Predicted ACE-Inhibitory Peptides from Chickpea Proteins on the ACE-I Binding Site

The three-dimensional (3D) structures of the predicted ACE-I inhibitory peptides were obtained from the PubChem or SATPdb databases and prepared for molecular docking (adding the polar hydrogens and assigning charges). Peptides without 3D structures available on PubChem or SATPdb databases were excluded from the molecular docking analysis. The crystallographic structure of human ACE-I in complex with the ACE-I inhibitor lisinopril (PDB ID: 1O86) [11] was obtained from the Protein DataBank (https://www.rcsb.org/) (accessed on 15 December 2021). The ACE-I structure was prepared for molecular docking by removing the ACE-I inhibitor lisinopril and the water molecules. The ACE-I model included polar hydrogens and charges [12]. Incomplete side chains were replaced using the Dunbrack 2010 rotamer library [13]. Docking was carried out using the flexible docking tool AutoDock Vina 1.1.2 [14,15]. The coordinates for docking in the ACE-I structure (PDB ID: 1O86) were x: 40.6559, y: 37.3827, and z: 43.3401 with a radius of 20 Å [14]. The selected area covers the active site of the ACE-I. The following parameters were established: The number of binding models per ligand was set to 10, the exhaustiveness of search was set to 8, and the maximum energy difference between models was set to 2 kcal/mol. The best docking pose for each predicted ACE-inhibitory peptide interacting with the active site of ACE-I was selected based on the binding-energy scores. Discovery Studio software v21.1.0 was used to visualize the dockings between CP and the active site of the ACE-I (hydrogen bonds, attractive charges, hydrophilic, and hydrophobic interactions). Predicted poses with nonfavorable interactions were discarded. Lisinopril was used as a control for the docking analysis. 

### 2.5. ADMET Prediction Analysis

ADMET is an important set of pharmacokinetics properties for identifying bioactive peptides with the potential to exert in vivo activity. In brief, ADMET properties of ACE-I inhibitory peptides with EC50 values <200 µM were predicted using the ADMETlab 2.0 server [15]. The tool “SMILES”, available in the BIOPEP-UWM database, was employed to translate the amino acid sequences [9]. Afterwards, SMILES sequences from ACE-I inhibitory peptides were loaded into the “ADMET Evaluation” tool available in the ADMETlab 2.0 server, which enables predicting 53 pharmacokinetics properties. The following ADMET properties were calculated for each of the ACE-I inhibitory peptides: (1) fulfillment of Lipinski rules (optimal values (OV): molecular weight <500 Da, logarithm of octanol-water partition coefficient <5, hydrogen bond donors <5, hydrogen bond acceptors <10); (2) human intestinal absorption (HI; OV > 30%); (3) human oral bioavailability (OV > 30%); (4) volume distribution (OV: 0.04–20 L/kg); (5) half-life (OV ≥ 0.5 h); and (6) rat oral acute toxicity (OV > 500 mg/kg). The ADMET characteristics of ACE-I inhibitory peptides were interpreted based on the criteria described in the ADMETlab 2.0 platform [15].

## 3. Results and Discussion

### 3.1. ACE-I Inhibitory Peptides Prediction from Chickpea Seed Legumin and Provicilin

In this study, the ACE-I inhibitory potential of legumin and provicilin peptides from chickpea were predicted as well as the ACE-I inhibitory peptides released by simulated enzymatic hydrolysis. Additionally, the potential interactions between the ACE-I inhibitory peptides and the active site of ACE-I were evaluated through molecular docking, and the potential pharmacokinetics of the peptides were assessed. The peptides with ACE-I inhibitory activity predicted by the “profiles of potential biological activity” tool within the sequences of legumin and provicilin are shown in Supplementary Material Table S1. A total of 216 and 165 peptides with ACE-I inhibitory activity were predicted in the chickpea legumin (A = 0.4335) and provicilin (A = 0.3642) sequences, respectively (Table 1). More than half of the peptides (57%) contained hydrophobic amino acids in the carboxyl-terminal position, such as proline, leucine, and tyrosine (Supplementary Material Table S1). Legumin and provicilin showed relatively high A values (0.4335 and 0.3634) compared with the proteins from other plant sources (rice prolamins A = 0.240, wheat prolamins A = 0.29, wheat γ-gliadin A = 0.304, oat 12S seed storage globulin A = 0.315, common sunflower 11S globulin seed storage protein G3 A = 0.324, broad bean legumin chain B A = 0.331, garden pea legumin J A = 0.334, soybean basic 7S subunit globulin A = 0.34, barley γ-hordein 1 A = 0.348, pumpkin 11S globulin A = 0.350, cocoa storage protein A = 0.351) [16]. The A values were even superior to some proteins from animal sources (red-necked wallaby a-lactalbumin A = 0.264, chicken tropomyosin 1 a chain A = 0.278, Arabian camel α-lactalbumin A = 0.309, horse α-lactalbumin A = 0.309, goat κ-casein A = 0.323, sheep α-lactalbumin A = 0.325, goat α-lactalbumin A = 0.325) [16]. B values (potential ACE-I inhibitory activity) of legumin and provicilin were 0.0201 and 0.0110, respectively (Table 1). Overall, both legumin and provicilin are potentially good sources of ACE-I inhibitory peptides. However, theoretically, the provicilin peptides are more potent ACE-I inhibitors than the legumin ones.

**Figure 2:**
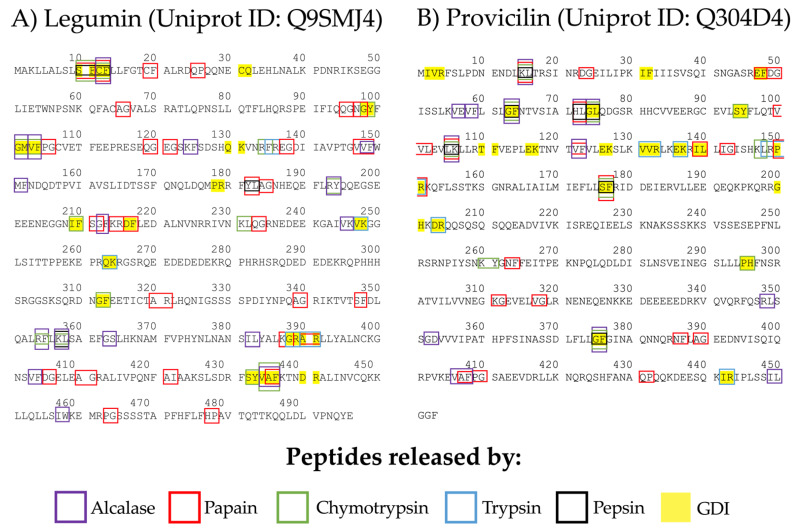
Profile of ACE-I inhibitory peptides released after in silico enzymatic hydrolysis of chickpea (*Cicer arietinum* L.) proteins. (**A**) legumin and (**B**) provicilin. The color indicates the enzyme that released the peptides after enzymatic hydrolysis. GID: Gastrointestinal digestion.

Papain and alcalase hydrolysates from different food sources could have antitumor, antioxidant, antidiabetic, and antihypertensive activities [17,18]. Thus, we analyzed the ACE-I inhibitory potential of peptides released by in silico papain and alcalase hydrolysis using the BIOPEP-UWM database. Furthermore, the ACE-I inhibitory peptides predicted to be released after hydrolysis of legumin and provicilin with human gastrointestinal enzymes were analyzed as well (pepsin, trypsin, chemotrypsin). Figure 2 shows the profiles of peptides with ACE-I inhibitory potential that were predicted to be released after enzymatic hydrolysis (for more information, see Supplementary Material Tables S2–S7). The papain, alcalase, pepsin, trypsin, and chymotrypsin hydrolysates accounted for a total of 162 ACE-I inhibitory peptides (legumin n = 87, provicilin n = 75), of which 59 were different peptides. All the peptides released from enzymatic hydrolysis were di and tripeptides (94.4% and 5.6%, respectively). ACE-I inhibitory peptides can be up to 12 amino acids long, but di and tripeptides have the highest ACE-I inhibitory potential [19]. For both legumin and provicilin, papain can release more ACE-I inhibitory peptides than pepsin, trypsin, alcalase, and chymotrypsin, with A_E_ values of 0.0605 and 0.0442, respectively. Contrarily, trypsin hydrolysates had the lowest A_E_ values (0.0101 for legumin and 0.0362 for provicilin), but the peptides released from legumin after trypsin hydrolysis had the most potent ACE-I inhibitory potential (B_E_ = 0.00018). Pepsin hydrolysis can release potent ACE-I inhibitory peptides from provicilin (B_E_ = 0.00011). Papain and alcalase hydrolysates of legumin and provicilin generated ACE-I inhibitory peptides (A_E_ = 0.0605–0.0442 and A_E_ = 0.0323–0.0287, respectively) at higher frequency than human proteases (Table 2). However, the simulated gastrointestinal digestion of legumin and provicilin generated ACE-I inhibitory peptides with a more potent inhibitory effect (B_E_ = 0.00018–0.00302) than the ones generated with papain (B_E_ = 0.00302–0.00139) and alcalase (B_E_ = 0.00273–0.00067). Previous studies have reported that lower ACE-I IC50 values are obtained after in vitro gastrointestinal digestion (140–229 µg/mL) than after in vitro papain and alcalase hydrolysis (180–282 µg/mL and 316–228 µg/mL, respectively) of two chickpea variates [8]. This means that ACE-I inhibitory peptides can be released during the gastrointestinal digestion of chickpea proteins. However, in vivo factors, such as epithelial peptidases, the mucus barrier, and intestinal absorption rate, among others, can limit the bioavailability and effectiveness of these inhibitory peptides [20].

### 3.2. Molecular Interaction of Chickpea (Cicer arietinum L.) ACE-I Inhibitory Peptides and ACE-I

Peptides predicted to be released by enzymatic hydrolysis with EC50 values < 200 µM were selected for molecular docking analysis with ACE-I (PDB ID: 1O86). Docking results are shown in Table 2. A total of 20 peptides were submitted for molecular docking analysis (ten from legumin, five from provicilin, and five for both proteins). Molecular docking analyses of all the peptides evaluated are available in an online open access repository (https://doi.org/10.6084/m9.figshare.19710343) (accessed on 4 May 2022). The binding energy between legumin or provicilin peptides with the ACE-I active sites ranges from −5.7 to −9.2 kcal/mol. The main molecular interactions between the ACE-I inhibitory peptides and the amino acid residues from the ACE-I active sites were conventional hydrogen bonds and attractive charges (distances ranges from 1.92 to 3.08 Å) (Supplemental Material Table S8). These findings suggest that chickpea peptides bind strongly and tightly to the active site of ACE-I, as molecular interactions via hydrogen bonds and attractive charges at short distances (<3.0 Å) aid in the stabilization of the complexes formed [19,21]. Three pockets forming the active sites of ACE-I have been reported: S1 (Ala354, Glu384, and Tyr523), S2 (Gln281, His353, Lys511, His513, and Tyr520) and S1’(Gln162) [22]. Peptides that interact with these residues have the potential to inhibit ACE-I by competitive inhibition [23]. In this sense, most legumin and provicilin peptides can interact through hydrogen bonds with the residues Ala354 and Glu384 from pocket S1 of the active site of ACE-I. These molecular interactions are less frequently with residues Gln281, Lys511, and Try520 from pocket S2, but all legumin and provicilin peptides interact with residues His353 and His513 from pocket S2 mainly through hydrogen bonds.

**Figure 3:**
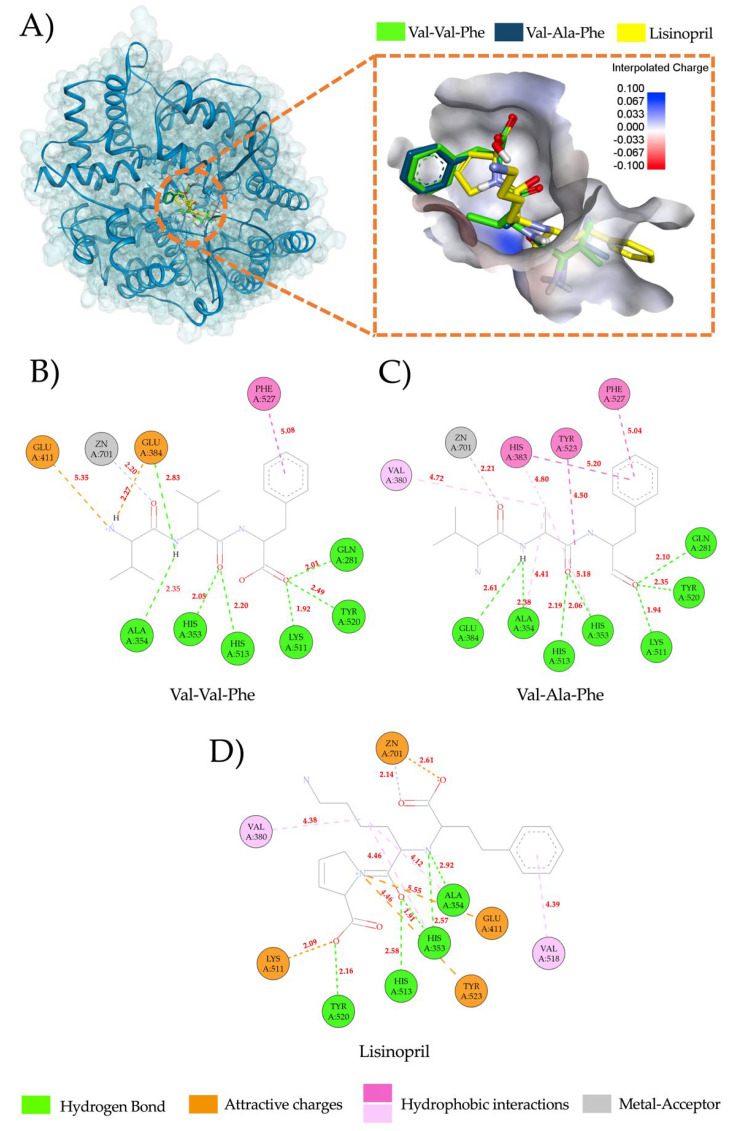
Docking modeling visualization of VVF and VAF tripeptide with the active site of ACE-I. (**A**) Overlap of docking results of VAF and VVF with ACE-I-lisinopril complex (PDB: 1O8A). The box is the close-up view of the overlap poses of VAF, VVF, and lisinopril interacting with the ACE-I pocket. The peptides are presented in the stick model. VVF (Val-Val-Phe): Green sticks; VAF (Val-Ala-Phe): Blue sticks; Lisinopril: Yellow sticks. (**B**) Docking modeling visualization of VVF with the active site of ACE-I. (**C**) Docking modeling visualization of VAF tripeptide with the active site of ACE-I. (**D**) Docking modeling visualization of Lisinopril with the active site of ACE-I. Interactions distances (Å) are shown in red numbers.

Figure 3 shows the overlap of the best poses of the tripeptides VVF (binding energy: −9.2 kcal/mol) and VAF (binding energy: −8.6 kcal/mol) with the crystal structure of the ACE-1-lisinopril complex. VVF and VAF peptides have a structure and docking pose similar to lisinopril (Figure 3A). VVF and VAF peptides interact with residues Ala354, Try523, and Glu384 from pocket S1 trough hydrogen bonds and attractive charges (Figure 3B,C). Regarding pocket S2, both peptides interact with all residues through hydrogen bonds (1.92 to 2.49 Å), similar to lisinopril (Figure 3D). In general, the main interactions of chickpea peptides with residues from pockets S1 and S2 were hydrogen bonds, with distances ranging from 1.92 to 3.08 Å. Other legumin and provicilin peptides interacted with pockets S1 and S2 via attractive charges (Pi-Pi and Pi-alkyl interactions), but the molecular interaction distances were greater (2.04 to 5.41 Å) than those found for VVF or VVF. Both VVF and VAF peptides interacted with Zn ion. This is of relevance since the simultaneous interaction of ACE-I inhibitors with pockets S1, S2 and Zn ion (II) is associated with potent ACE-I inhibitory activity [24]. This could explain the potent binding of VVF and VAF with ACE-I that was observed in docking analyses. Notably, ACE-I drug inhibitors, such as lisinopril, captopril, and enalapril, tightly interact with ACE-I residues His353, Tyr520, Ala354, Glu384, and His513 [25,26,27], which is in line with the molecular docking interactions of CP shown in the present study (Supplemental Material Table S8). Overall, both CP and ACE-I drug inhibitors had similar molecular interactions with the active site of ACE-I.

### 3.3. ADMET Analyses Showed That CP Are Bioavailable and Non-Toxic

The ADMET properties of ACE-I inhibitory peptides with an EC50 < 200 uM were screened using the ADMETlab 2.0 platform [15]. Compliance with the Lipinski rules is desirable for attributing biological activity to a compound once it is ingested by human beings. Compounds that fulfill the Lipinski rules must meet the following criteria: (1) molecular weight < 500 Da, (2) logarithm of octanol-water partition coefficient < 5, (3) hydrogen bond donors < 5 and (4) hydrogen bond acceptors <10 [28]. Notably, all ACE-I inhibitory peptides derived from enzymatic hydrolysis of CP fulfilled the Lipinski rules, which suggests good potential to exert their function in vivo (Supplementary Material Table S9). Human intestinal absorption (HIA) and oral bioavailability are the most important pharmacokinetic properties in predicting the biological activity of compounds that are delivered via the oral route [15]. In the present study, most peptides showed optimal values for HIA (75.0%, 15 out of 20), and 85.0% of peptides were more than 30% available in the blood circulation in normal conditions (HIA > 30%, 17 out of 20). Interestingly, almost all CP showed better values of HIA and human oral bioavailability than the obtained from lisinopril using the same ADMET analysis. These results are in line with previous in vivo studies that have reported poor intestinal absorption and oral bioavailability of lisinopril [29,30]. Similarly, volume distribution was optimal (0.249–0.819 L/kg) for all the peptides, which means that they can be distributed in systemic circulation. Regarding half-life times, all peptides had a short half-life (0.39–0.90 h), but this parameter is similar to the one reported for lisinopril and captopril and other ACE-I inhibitory peptides from other sources using ADMET analysis (0.70 and 0.86 h, respectively) [31]. ACE-I inhibitor drugs with short half-life times in serum are not uncommon [32], and their hypotensive effects can last up to hours after their consumption. This can be attributed to the capacity of ACE-I inhibitors to form reversible complexes with plasma proteins, which can serve as reservoirs of the drug [33]. Notably, food-derived antihypertensive peptides have shown hypotensive effects in vivo that can last hours, similar to ACE-I drug inhibitors [34,35]. It should be highlighted that the peptides VVF and VAF, which interact with Zn ion, showed optimal ADMET properties, suggesting their potential to exert their effect in vivo. The ACE-I inhibitory activity of these peptides has been proven in vitro [36,37], but data about their hypotensive effects in vivo remain unknown. Considering that almost all CP (95.0%, 19 out of 20) showed optimal values on rat oral acute toxicity analysis (>500 mg/kg), they can be considered potentially nontoxic peptides. Overall, the results encourage the assessment in vivo of the bioavailability and the antihypertensive effect of ACE-I inhibitory peptides from chickpea proteins and their use as an ingredient for the formulation of functional foods.

## 4. Conclusions

Papain, alcalase, pepsin, trypsin, and chymotrypsin hydrolysates of legumin and provicilin from chickpea are potential sources of ACE-I inhibitory peptides. The molecular docking interactions of the ACE-I inhibitory peptides from chickpea proteins suggest strong interactions with residues from the active sites of ACE-I through hydrogen bonds and salt bridges. Furthermore, ADMET prediction analysis indicates that CP are potentially absorbed at the intestinal level, are bioavailable, are adequately distributed in systemic circulation, and are nontoxic. Further studies evaluating the antihypertensive effect of chickpea hydrolysates in vivo are warranted. The use of databases and in silico assessments as the first steps in the search for bioactive peptides derived from food sources could reduce time and optimize resources in research laboratories.

## Figures and Tables

**Table 1:** ACE-I inhibitory predicted values of legumin and provicilin from chickpea (*Cicer arietinum* L.).

Protein	A	B	In Silico Hydrolysis		
			Proteases	A_E_	B_E_
Legumin	0.4335	0.0201	Pepsin	0.0101	0.00112
			Trypsin	0.0101	0.00018
			Chymotrypsin	0.0282	0.00168
			Gastrointestinal digestion	0.0343	0.00206
			Papain	0.0605	0.00302
			Alcalase	0.0323	0.00273
Provicilin	0.3642	0.0110	Pepsin	0.0155	0.00011
			Trypsin	0.0132	0.00058
			Chymotrypsin	0.0221	0.00035
			Gastrointestinal digestion	0.0419	0.00024
			Papain	0.0442	0.00139
			Alcalase	0.0287	0.00067

**Table 2:** The molecular docking results for CP with the active sites of ACE-I.

Peptide/Ligand	BIOPEP ID	Binding Energy (Kcal/Mol)	Protein	Location	Released by	EC50 (µM/L)	PubChem/Satpdb ID
Lisinopril		−8.6					5362119
VVF	9044	−9.2	Legumin	[147–149]	Papain	35.45	7014911
VAF	8126	−8.6	Legumin	[434–436]	GID; Chymotrypsin (A)	35.8	satpdb14951
				[406–408] [434–436]	Alcalase		
IW	7544	−8.5	Legumin	[457,458]	Alcalase	4.7	7019084
RY	3380	−8.4	Legumin	[193,194]	Chymotrypsin (A); Alcalase	10.5	7021456
RF	3489	−8.2	Legumin	[134,135]	Chymotrypsin (A)	93	150964
				[354,355]	Chymotrypsin (A); Pepsin (pH 1.3);Alcalase		
IVR	7502	−8.0	Provicilin	[2–4]	GID	0.81	25217595
YL	3350	−7.9	Legumin	[182–183]	Papain; GID	122	87071
VF	3384	−7.8	Legumin	[103,104]	Chymotrypsin (A); GID; Alcalase	9.2	6993120
				[148,149] [403,404]	Alcalase		
			Provicilin	[122,123]	Papain; Alcalase		
				[58,59]	Alcalase		
SF	7685	−7.7	Provicilin	[176,177]	Papain; Chymotrypsin (A), Pepsin (pH 1.3); GID	130.2	7009597
			Legumin	[10,11]	Papain; Chymotrypsin (A), Pepsin (pH 1.3); GID		
				[347,348]	Papain		
AF	7583	−7.5	Provicilin	[407,408]	Papain	190	6992394
			Legumin	[435,436]	Papain		
KF	7692	−7.4	Legumin	[124,125]	Alcalase	28.3	151410
CF	7751	−7.3	Legumin	[12,13]	Papain; Chymotrypsin (A), Pepsin (pH 1.3); GID; Alcalase	1.96	25051327
				[19,20]	Papain		
PR	3537	−7.2	Provicilin	[150,151]	Papain; Trypsin; GID	4.1	151004
			Legumin	[178,179]	GID		
TF	8185	−7.1	Provicilin	[110,111]	GID	18	7010580
DR	10091	−7.0	Provicilin	[203,204]	Trypsin; GID	110.5	16122509
			Legumin	[440,441]	GID		
LR	9213	−6.8	Provicilin	[148,149]	Trypsin	158	152914
IL	9079	−6.5	Provicilin	[139,140]	Papain; GID	54.95	7019083
				[449,450]	Alcalase		
			Legumin	[382,383]	Alcalase		
AR	7742	−6.4	Legumin	[390,391]	GID; Trypsin; Papain	95.5	446132
				[320,321]	Papain		
DG	7681	−5.8	Provicilin	[23,24]	Papain	190.1410	151148
				[49,50]	Papain		
				[405,406]	Papain		
VK	7558	−5.7	Legumin	[247,248]	Trypsin; GID	13	168058
				[245,246]	Alcalase		

## Data Availability

Data are available within the article, supplementary materials, and data repository (https://doi.org/10.6084/m9.figshare.19710343) (accessed on 4 May 2022).

## Supplementary Materials

The following supporting information can be downloaded at: https://www.mdpi.com/article/10.3390/foods11111576/s1, Table S1. General profile of fragments with ACE inhibitory activity from legumin and provicilin proteins from chickpea (*Cicer arietinum* L.); Table S2. Peptide release from legumin and provicilin proteins after trypsin hydrolysis; Table S3. Peptide release from legumin and provicilin proteins after pepsin hydrolysis; Table S4. Peptide release from legumin and provicilin proteins after chymotrypsin hydrolysis; Table S5. Peptide release from legumin and provicilin proteins after gastrointestinal digestion hydrolysis; Table S6. Peptide release from legumin and provicilin proteins after papain hydrolysis; Table S7. Peptide release from legumin and provicilin proteins after alcalase hydrolysis; Table S8. Molecular docking interactions of chickpea peptides and active sites of ACE-I; Table S9. ADMET characteristics of ACE-I inhibitory peptides with EC50 < 200 µM from chickpea proteins.
