# Supplementary material for: Prediction of ACE-I Inhibitory Peptides Derived from Chickpea (Cicer arietinum L.): In Silico Assessments Using Simulated Enzymatic Hydrolysis, Molecular Docking and ADMET Evaluation
Source: Foods. 2022 May 27;11(11):1576. doi: 10.3390/foods11111576 (PMC9180818; doi:10.3390/foods11111576)
Supplement: Supplementary file 1 [file foods-11-01576-s001.zip › foods-1735173-supplementary.pdf]

Supplementary material

Table S1. General profile of fragments with ACE inhibitory activity from legumin and provicilin proteins from chickpea (Cicer arietinum L.)

		**Legumin**		**Provicilin**	
**ID**	**Sequence**	Repetition	Location	Repetition	Location
3257	RL	2	[321-322],[391-392]	3	[133-134],[348-349],[417-418]
3258	IR	-	-	1	[442-443]
3361	LKL	1	[356-358]	2	[14-16],[105-107]
3370	AVP	1	[142-144]	-	-
3380	RY	1	[193-194]	-	-
3381	LY	2	[383-384],[393-394]	-	-
3383	IY	1	[334-335]	1	[256-257]
3384	VF	3	[103-104],[148-149],[403-404]	2	[58-59],[122-123]
3385	MF	2	[151-152],[370-371]	-	-
3406	TAP	1	[469-471]	-	-
3489	RF	4	[134-135],[180-181],[354-355],[430-431]	2	[4-5],[344-345]
3494	HY	1	[374-375]	-	-
3502	FP	1	[104-105]	1	[408-409]
3507	IPA	1	[357-359]	1	[357-359]
3532	GY	1	[98-99]	-	-
3537	PR	3	[114-115],[178-179],[261-262]	1	[150-151]
3542	LQP	1	[74-76]	-	-
3543	LRP	1	[148-150]	1	[148-150]
3550	YL	1	[182-183]	-	-
3551	LF	2	[15-16],[475-476]	1	[371-372]
3553	YG	1	[261-262]	1	[261-262]
3556	FY	1	[181-182]	-	-
3573	AFP	1	[407-409]	1	[407-409]
3713	LLP	1	[293-295]	1	[293-295]
7502	IVR	1	[2-4]	1	[2-4]
7513	PL	2	[114-115],[445-446]	2	[114-115],[445-446]
7544	IW	1	[457-458]	-	-
7549	LKP	1	[39-41]	-	-
7558	VK	2	[245-246],[247-248]	2	[219-220],[403-404]
7562	IA	3	[141-142],[160-161],[422-423]	2	[69-70],[166-167]
7581	IP	3	[28-29],[357-358],[444-445]	3	[28-29],[357-358],[444-445]
7582	RP	1	[462-463]	2	[149-150],[401-402]
7583	AF	1	[435-436]	1	[407-408]
7584	AP	1	[470-471]	-	-
7585	LA	2	[5-6],[183-184]	1	[388-389]
7586	KR	4	[214-215],[264-265],[278-279],[294-295]	1	[137-138]

7587	VP	4	[143-144],[372-373],[416-417],[491-492]	-	-
7588	RA	4	[71-72],[389-390],[412-413],[441-442]	2	[163-164],[300-301]
7589	YA	2	[384-385],[394-395]	-	-
7590	AA	1	[423-424]	-	-
7591	GF	2	[212-213],[312-313]	3	[63-64],[375-376],[452-453]
7592	FR	1	[135-136]	1	[177-178]
7593	IF	2	[91-92],[209-210]	1	[31-32]
7594	VG	1	[317-318]	1	[317-318]
7595	IG	1	[326-327]	1	[142-143]
7596	GI	3	[50-51],[143-144],[377-378]	3	[5--51],[143-144],[377-378]
7597	GM	1	[101-102]	-	-
7598	GA	1	[242-243]	1	[43-44]
7599	GL	2	[50-51],[250-251]	2	[74-75],[318-319]
7600	AG	4	[65-66],[184-185],[339-340],[410-411]	1	[389-390]
7601	GH	1	[200-201]	1	[200-201]
7602	HL	1	[35-36]	1	[72-73]
7603	GR	4	[234-235],[340-341],[388-389],[411-412]	-	-
7604	KG	4	[241-242],[248-249],[387-388],[399-400]	1	[311-312]
7605	FG	3	[16-17],[100-101],[362-363]	1	[376-377]
7606	DA	1	[220-221]	-	-
7607	GS	7	[122-123],[198-199],[266-267],[304-305],[327-328],[363-364],[464-465]	3	[78-79],[290-291],[410-411]
7608	GV	2	[66-67],[146-147]	-	-
7611	GK	1	[310-311]	1	[31--311]
7612	GT	1	[17-18]	-	-
7615	GE	2	[120-121],[406-407]	3	[24-25],[312-313],[390-391]
7616	GG	4	[49-50],[206-207],[249-250],[303-304]	1	[451-452]
7617	QG	3	[95-96],[119-120],[233-234]	-	-
7618	SG	1	[211-212]	2	[160-161],[351-352]
7619	LG	4	[62-63],[73-74],[374-375],[450-451]	4	[62-63],[73-74],[374-375],[450-451]
7620	GD	1	[138-139]	1	[352-353]
7621	TG	1	[145-146]	-	-
7622	EG	5	[48-49],[121-122],[137-138],[197-198],[205-206]	2	[289-290],[309-310]
7623	EA	1	[409-410]	1	[214-215]
7624	NG	2	[97-98],[311-312]	1	[42-43]
7625	PG	2	[105-106],[463-464]	1	[409-410]

7628	VR	2	[3-4],[132-133]	2	[3-4],[132-133]
7644	ITT	1	[253-255]	-	-
7648	FCF	1	[11-13]	-	-
7649	LRY	1	[192-194]	-	-
7654	NKL	1	[230-232]	-	-
7680	QK	3	[130-131],[263-264],[448-449]	3	[193-194],[434-435],[440-441]
7681	DG	1	[405-406]	3	[23-24],[49-50],[77-78]
7683	NF	1	[419-420]	2	[263-264],[386-387]
7684	SY	1	[432-433]	1	[94-95]
7685	SF	3	[10-11],[169-170],[347-348]	1	[176-177]
7691	KY	1	[260-261]	1	[260-261]
7692	KF	1	[124-125]	-	-
7693	KL	4	[3-4],[231-232],[357-358],[450-451]	3	[15-16],[1-6-1-7],[147-148]
7698	NK	2	[59-60],[230-231]	2	[259-260],[327-328]
7741	RR	2	[179-180],[226-227]	1	[198-199]
7742	AR	2	[320-321],[390-391]	-	-
7751	CF	2	[12-13],[19-20]	-	-
7807	LLF	1	[14-16]	-	-
7810	KP	1	[40-41]	1	[194-195]
7823	FAL	1	[20-22]	-	-
7826	EI	1	[90-91]	4	[25-26],[182-183],[266-267],[286-287]
7827	IE	1	[52-53]	3	[171-172],[183-184],[226-227]
7828	EV	6	[57-58],[91-92],[103-104],[313-314],[405-406],[414-415]	6	[57-58],[91-92],[103-104],[313-314],[405-406],[414-415]
7829	VE	1	[108-109]	5	[56-57],[85-86],[112-113],[285-286],[314-315]
7831	LQ	5	[74-75],[80-81],[232-233],[350-351],[452-453]	3	[75-76],[97-98],[275-276]
7832	LN	4	[36-37],[222-223],[377-378],[396-397]	1	[282-283]
7833	PT	1	[144-145]	-	-
7834	TQ	1	[481-482]	-	-
7836	PP	1	[256-257]	-	-
7837	PQ	3	[26-27],[337-338],[417-418]	2	[273-274],[432-433]
7840	EK	4	[240-241],[258-259],[277-278],[293-294]	4	[116-117],[126-127],[136-137],[270-271]
7841	KE	2	[259-260],[459-460]	3	[135-136],[329-33-],[404-405]
7842	HP	1	[477-478]	1	[361-362]
7843	PH	3	[281-282],[297-298],[373-374]	1	[295-296]
7844	HK	1	[366-367]	2	[146-147],[201-202]
8126	VAF	1	[434-436]	1	[406-408]
8185	TF	2	[82-83],[110-111]	1	[110-111]
8193	AI	2	[243-244],[421-422]	1	[167-168]
8382	RYQ	1	[193-195]	-	-

8513	FVP	1	[371-373]	-	-
8951	AV	3	[142-143],[161-162],[479-480]	-	-
9031	LEE	1	[188-190]	1	[188-190]
9036	YVA	1	[433-435]	-	-
9044	VVF	1	[147-149]	-	-
9046	VQV	1	[340-342]	1	[340-342]
9064	LEK	2	[115-117],[125-127]	2	[115-117],[125-127]
9073	TP	2	[157-158],[255-256]	1	[268-269]
9074	DF	1	[216-217]	-	-
9076	FQ	1	[170-171]	1	[345-346]
9077	YV	1	[433-434]	-	-
9078	YE	1	[495-496]	-	-
9079	IL	1	[382-383]	5	[26-27],[139-140],[168-169],[304-305],[449-450]
9090	WM	1	[150-151]	-	-
9173	RG	2	[265-266],[302-303]	2	[88-89],[199-200]
9184	ST	1	[468-469]	1	[157-158]
9185	YN	2	[335-336],[375-376]	-	-
9211	FKR	1	[213-215]	-	-
9213	LR	3	[22-23],[192-193],[353-354]	4	[108-109],[148-149],[250-251],[319-32-]
9553	QLDL	1	[487-490]	-	-
9566	QP	4	[25-26],[75-76],[280-281],[296-297]	1	[431-432]
9729	VVR	1	[131-133]	1	[131-133]
9731	VVL	1	[100-102]	1	[100-102]
9742	EKR	2	[277-279],[293-295]	1	[136-138]
9754	NLR	1	[249-251]	1	[249-251]
9942	EF	2	[190-191],[361-362]	2	[47-48],[172-173]
9944	ER	2	[87-88],[184-185]	2	[87-88],[184-185]
10004	LQL	1	[452-454]	-	-
10020	YNL	1	[375-377]	-	-
10027	FF	1	[264-265]	1	[264-265]
10091	DR	2	[429-430],[440-441]	3	[203-204],[337-338],[416-417]
10092	LP	2	[7-8],[294-295]	2	[7-8],[294-295]

Table S2. Peptide release from legumin and provicilin proteins after trypsin hydrolysis.

Protein	Peptide ID	Sequence	Chemical mass	EC50 uM	Location
Provicilin	3258	IR	287.3480	695	[442-443]
Provicilin	3537	PR	271.3050	4.1	[150-151]
Legumin	7558	VK	245.3070	13	[247-248]
Legumin	7592	FR	321.3650	920	[135-136]
Legumin	7603	GR	231.2400	3200	[388-389]
Legumin	7680	QK	274.3050	885	[263-264]
Legumin	7742	AR	245.2670	95.5	[390-391]
Provicilin	7840	EK	275.2900	0	[136-137]
Provicilin	9213	LR	287.3480	158	[148-149]
Provicilin	9729	VVR	372.4540	249.7	[131-133]
Provicilin	10091	DR	289.2770	110.5	[203-204]

Table S3. Peptide release from legumin and provicilin proteins after pepsin hydrolysis.

Protein	Peptide ID	Sequence	Chemical mass	EC50 uM	Location
Legumin	3489	RF	321.3650	93	[354-355]
Legumin	3550	YL	294.3300	122	[182-183]
Legumin	7685	SF	252.2570	130.2	[10-11]
Legumin	7693	KL	259.3340	50.2	[357-358]
Legumin	7751	CF	285.1770	1.96	[12-13]
Provicilin	7591	GF	222.2290	630	[63-64]
Provicilin	7591	GF	222.2290	630	[375-376]
Provicilin	7599	GL	188.2120	2500	[74-75]
Provicilin	7602	HL	268.3020	3200	[72-73]
Provicilin	7685	SF	252.2570	130.2	[176-177]
Provicilin	7693	KL	259.3340	50.2	[15-16]
Provicilin	7693	KL	259.3340	50.2	[106-107]

Table S4. Peptide release from legumin and provicilin proteins after chymotrypsin hydrolysis.

Protein	Peptide ID	Sequence	Chemical mass	EC50 uM	Location
Legumin	3380	RY	337.3580	10.5	[193-194]
Legumin	3384	VF	264.3100	9.2	[103-104]
Legumin	3489	RF	321.3650	93	[134-135]
Legumin	3489	RF	321.3650	93	[354-355]
Legumin	3532	GY	238.2220	210	[98-99]
Legumin	7591	GF	222.2290	630	[312-313]
Legumin	7593	IF	278.3370	930	[209-210]
Legumin	7597	GM	206.2500	1400	[101-102]
Legumin	7684	SY	268.2500	66.3	[432-433]
Legumin	7685	SF	252.2570	130.2	[10-11]
Legumin	7693	KL	259.3340	50.2	[231-232]
Legumin	7693	KL	259.3340	50.2	[357-358]
Legumin	7751	CF	285.1770	1.96	[12-13]
Legumin	8126	VAF	335.3890	35.8	[434-436]
Provicilin	7591	GF	222.2290	630	[63-64]
Provicilin	7591	GF	222.2290	630	[375-376]
Provicilin	7599	GL	188.2120	2500	[74-75]
Provicilin	7684	SY	268.2500	66.3	[94-95]
Provicilin	7685	SF	252.2570	130.2	[176-177]
Provicilin	7691	KY	309.3440	13	[260-261]
Provicilin	7693	KL	259.3340	50.2	[15-16]
Provicilin	7693	KL	259.3340	50.2	[106-107]
Provicilin	7693	KL	259.3340	50.2	[147-148]
Provicilin	7843	PH	252.2590	0	[295-296]

Table S5. Peptide release from legumin and provicilin proteins after simulated gastrointestinal digestion.

Protein	Peptide ID	Sequence	Chemical mass	EC50 uM	Location
Legumin	3384	VF	264.3100	9.2	[103-104]
Legumin	3532	GY	238.2220	210	[98-99]
Legumin	3537	PR	271.3050	4.1	[178-179]
Legumin	7558	VK	245.3070	13	[247-248]
Legumin	7591	GF	222.2290	630	[312-313]
Legumin	7593	IF	278.3370	930	[209-210]
Legumin	7597	GM	206.2500	1200	[101-102]
Legumin	7603	GR	231.2400	3200	[388-389]
Legumin	7680	QK	274.3050	885	[130-131]
Legumin	7680	QK	274.3050	885	[263-264]
Legumin	7684	SY	268.2500	66.3	[432-433]
Legumin	7685	SF	252.2570	130.2	[10-11]
Legumin	7742	AR	245.2670	95.5	[390-391]
Legumin	7751	CF	285.1770	1.96	[12-13]
Legumin	8126	VAF	335.3890	35.8	[434-436]
Legumin	9074	DF	280.2660	363.1	[216-217]
Legumin	10091	DR	289.2770	110.5	[440-441]
Provicilin	3258	IR	287.3480	695	[442-443]
Provicilin	3537	PR	271.3050	4.1	[150-151]
Provicilin	7502	IVR	386.4810	0.81	[2-4]
Provicilin	7591	GF	222.2290	630	[63-64]
Provicilin	7591	GF	222.2290	630	[375-376]
Provicilin	7593	IF	278.3370	930	[31-32]
Provicilin	7599	GL	188.2120	2500	[74-75]
Provicilin	7601	GH	212.1940	3100	[200-201]
Provicilin	7684	SY	268.2500	66.3	[94-95]
Provicilin	7685	SF	252.2570	130.2	[176-177]
Provicilin	7840	EK	275.2900	-	[116-117]
Provicilin	7840	EK	275.2900	-	[126-127]
Provicilin	7840	EK	275.2900	-	[136-137]
Provicilin	7843	PH	252.2590	-	[295-296]
Provicilin	8185	TF	266.2820	18	[110-111]
Provicilin	9079	IL	244.3200	54.95	[139-140]
Provicilin	9729	VVR	372.4540	249.7	[131-133]
Provicilin	9942	EF	294.2930	-	[47-48]
Provicilin	10091	DR	289.2770	110.5	[203-204]

Table S6. Peptide release from legumin and provicilin proteins after papain hydrolysis.

Protein	Peptide ID	Sequence	Chemical mass	EC50 uM	Location
Legumin	3550	YL	294.3300	122	[182-183]
Legumin	7583	AF	236.2560	190	[435-436]
Legumin	7586	KR	302.3620	380	[214-215]
Legumin	7600	AG	146.1310	2500	[65-66]
Legumin	7600	AG	146.1310	2500	[184-185]
Legumin	7600	AG	146.1310	2500	[339-340]
Legumin	7600	AG	146.1310	2500	[410-411]
Legumin	7604	KG	203.2260	3200	[387-388]
Legumin	7617	QG	203.1830	7400	[95-96]
Legumin	7617	QG	203.1830	7400	[119-120]
Legumin	7617	QG	203.1830	7400	[233-234]
Legumin	7618	SG	162.1320	8500	[211-212]
Legumin	7622	EG	204.1680	10000	[121-122]
Legumin	7622	EG	204.1680	10000	[137-138]
Legumin	7624	NG	189.1560	12000	[97-98]
Legumin	7625	PG	172.1690	17000	[105-106]
Legumin	7625	PG	172.1690	17000	[463-464]
Legumin	7681	DG	190.1410	12.3	[405-406]
Legumin	7685	SF	252.2570	130.2	[10-11]
Legumin	7685	SF	252.2570	130.2	[347-348]
Legumin	7693	KL	259.3340	50.2	[357-358]
Legumin	7742	AR	245.2670	95.5	[320-321]
Legumin	7742	AR	245.2670	95.5	[390-391]
Legumin	7751	CF	285.1770	1.96	[12-13]
Legumin	7751	CF	285.1770	1.96	[19-20]
Legumin	7842	HP	252.2590	NR	[477-478]
Legumin	8193	AI	202.2390	3.41	[421-422]
Legumin	9044	VVF	363.4430	35.45	[147-149]
Legumin	9074	DF	280.2660	363.1	[216-217]
Legumin	9566	QP	243.2480	598.1	[25-26]
Provicilin	3384	VF	264.3100	9.2	[122-123]
Provicilin	3537	PR	271.3050	4.1	[150-151]
Provicilin	7583	AF	236.2560	190	[407-408]
Provicilin	7594	VG	174.1850	1100	[317-318]
Provicilin	7595	IG	188.2120	1200	[142-143]
Provicilin	7600	AG	146.1310	2500	[389-390]
Provicilin	7602	HL	268.3020	3200	[72-73]
Provicilin	7604	KG	203.2260	3200	[311-312]
Provicilin	7625	PG	172.1690	3700	[409-410]
Provicilin	7681	DG	190.1410	12.3	[23-24]
Provicilin	7681	DG	190.1410	12.3	[49-50]
Provicilin	7683	NF	279.2810	46.3	[263-264]
Provicilin	7683	NF	279.2810	46.3	[386-387]
Provicilin	7685	SF	252.2570	130.2	[176-177]
Provicilin	7693	KL	259.3340	50.2	[15-16]
Provicilin	7693	KL	259.3340	50.2	[106-107]
Provicilin	9079	IL	244.3200	54.95	[139-140]
Provicilin	9566	QP	243.2480	598.1	[431-432]

Provicilin	9731	VVL	329.4260	0	[100-102]
Provicilin	9942	EF	294.2930	0	[47-48]

Table S7. Peptide release from legumin and provicilin proteins after alcalase hydrolysis.

Protein	Peptide ID	Sequence	Chemical mass	EC50 uM	Location
Legumin	3380	RY	337.3580	10.5	[193-194]
Legumin	3384	VF	264.3100	9.2	[103-104]
Legumin	3384	VF	264.3100	9.2	[148-149]
Legumin	3384	VF	264.3100	9.2	[403-404]
Legumin	3385	MF	296.3750	45	[151-152]
Legumin	3489	RF	321.3650	93	[354-355]
Legumin	7544	IW	317.3730	4.7	[457-458]
Legumin	7558	VK	245.3070	13	[245-246]
Legumin	7591	GF	222.2290	630	[212-213]
Legumin	7597	GM	206.2500	1400	[101-102]
Legumin	7607	GS	162.1320	3800	[363-364]
Legumin	7692	KF	293.3510	28.3	[124-125]
Legumin	7693	KL	259.3340	50.2	[357-358]
Legumin	7751	CF	285.1770	1.96	[12-13]
Legumin	8126	VAF	335.3890	35.8	[434-436]
Legumin	9079	IL	244.3200	54.95	[382-383]
Provicilin	3257	RL	287.3480	2439	[348-349]
Provicilin	3384	VF	264.3100	9.2	[58-59]
Provicilin	3384	VF	264.3100	9.2	[122-123]
Provicilin	7591	GF	222.2290	630	[63-64]
Provicilin	7591	GF	222.2290	630	[375-376]
Provicilin	7599	GL	188.2120	2500	[74-75]
Provicilin	7602	HL	268.3020	3200	[72-73]
Provicilin	7620	GD	190.1410	9200	[352-353]
Provicilin	7693	KL	259.3340	50.2	[15-16]
Provicilin	7693	KL	259.3340	50.2	[106-107]
Provicilin	7829	VE	246.2490	0	[56-57]
Provicilin	8126	VAF	335.3890	35.8	[406-408]
Provicilin	9079	IL	244.3200	54.95	[449-450]

Table S8. Molecular docking interactions of chickpea peptides and active sites of ACE-I.

Peptide	BIOPEP ID	Energy (kcal/mol)	S1			S2					S1'	Zn
			Ala 354	Glu 384	Tyr 523	Gln 281	His 353	Lys 511	His 513	Tyr 520	Glu 162	
VVF	9044	-9.2	V1 - Hydrogen bond (3.35 Å) V2 - Hydrogen bond (2.35 Å)	V1 - Salt bridge (2.27 Å) V2 - Hydrogen bond (2.83 Å)	Van der Waals	F3 - Hydrogen bond (2.01 Å)	V2 - Hydrogen bond (2.05 Å)	F3 - Hydrogen bond (1.92 Å)	V2 - Hydrogen bond (1.92 Å)	F3 - Hydrogen bond (2.49 Å)	N/A	V1- Metal-Acceptor (2.20 Å)
VAF	8126	-8.6	V1 - Hydrogen bond (3.42 Å) A2 - Hydrogen bond (2.38 Å)	A2 - Hydrogen bond (2.61 Å)	A2 - Amide-Pi Stacked (4.50 Å)	F3 - Hydrogen bond (2.10 Å)	A2- Hydrogen bond (2.06 Å)	F3 - Hydrogen bond (1.94 Å)	A2- Hydrogen bond (2.19 Å)	F3 - Hydrogen bond (2.35 Å)	N/A	V1- Metal-Acceptor (2.21 Å)
IW	7544	-8.5	I1- Two Hydrogen bonds (2.66 and 3.02 Å)	I1 - Salt bridge (1.94 Å)	Van der Waals	W2 - Hydrogen bond (2.09 Å)	I1 - Hydrogen bond (2.18 Å)	W2 - Salt bridge (2.12 Å)	I1 - Hydrogen bond (2.56 Å)	W2 - Hydrogen bond (2.39 Å)	N/A	Van der Waals
RY	3380	-8.4	R1- Hydrogen bond (2.40 Å)	R1 - Attractive charge (2.15 Å)	Van der Waals	Y2 - Hydrogen bond (2.04 Å)	R1 - Hydrogen bond (2.29 Å)	Y2- Hydrogen bond (1.94 Å)	R1- Hydrogen bond (2.27 Å)	Y2- Hydrogen bond (2.35 Å)	R1 - Hydrogen bond (2.19 Å) R1- Attractive	Van der Waals

											charge (4.60 Å)	
RF	3489	-8.2	R1 - Hydrogen bond (2.98 Å) F2- Two Hydrogen bonds (2.54 and 2.19 Å)	R1 - Three Attractive charges (2.25, 2.47, and 4.76 Å)	F2- Pi-Pi Stacked (3.97 Å)	Van der Waals	R1 - Hydrogen bond (2.25 Å)	N/A	F2- Hydrogen bond (2.25 Å)	Van der Waals	N/A	Van der Waals
IVR	7502	-8.0	N/A	N/A	Van der Waals	I1- Hydrogen bond (2.62 Å) V2- Hydrogen bond (2.06 Å)	V2- Pi-Alkyl (4.34 Å)	V2- Hydrogen bond (2.35 Å)	R3- Hydrogen bond (2.50 Å)	R3- Hydrog en bond (2.26 Å)	Van der Waals	N/A
YL	3350	-7.9	Y1- Hydrogen bond (2.24 Å) L2- Hydrogen bond (2.88 Å)	Y1- Attractive charge (2.57 Å) L2- Hydrogen bond (2.29 Å)	Van der Waals	N/A	Y1- Attractive charge (4.19 Å) Y1- Hydrogen bond (2.25 Å) L2- Pi-Alkyl (5.41)	N/A	Y1- Hydrogen bond (2.43 Å)	N/A	N/A	Van der Waals

VF	3384	-7.8	V1- Hydrogen bond (2.50 Å) F2- Hydrogen bond (2.72 Å)	V1- Two Attractive charges (2.44 and 4.97) F2- Hydrogen bond (2.16 Å)	F2- Pi-Pi Stacked (3.95 Å)	Van der Waals	V1- Hydrogen bond (2.20 Å)	N/A	V1- Hydrogen bond (2.31 Å)	Van der Waals	N/A	Van der Waals
SF	7685	-7.7	S1- Hydrogen bond (2.37 Å) F2- Hydrogen bond (2.85 Å)	S1- Two attractive charges (2.04 and 2.87 Å) F2- Hydrogen bond (2.10 Å)	F2- Pi-Pi Stacked (3.96 Å)	Van der Waals	S1- Hydrogen bond (2.23 Å)	N/A	S1- Hydrogen bond (2.31 Å)	Van der Waals	N/A	Van der Waals
AF	7583	-7.5	A1- Hydrogen bond (2.42 Å)	A1- Attractive charge (2.30 Å)	Van der Waals	Van der Waals	A1- Hydrogen bond (2.29 Å)	F2- Attractive charge (1.94 Å)	A1- Hydrogen bond (2.29 Å)	F2- Hydrog en bond (2.33 Å)	N/A	N/A
KF	7692	-7.4	K1 - Hydrogen bond (2.85 Å) F2 - Hydrogen bond (2.59 Å)	K1 – Two Salt bridges (2.22 and 2.84 Å)	F2 – Pi-Pi Stacked (3.99 Å)	N/A	K1 - Hydrogen bond (2.21 Å)	N/A	K1 - Hydrogen bond (2.25 Å)	Van der Waals	N/A	Van der Waals
CF	7751	-7.3	C1- Hydrogen bonds (2.44 Å) F2- Two Hydrogen bonds	C1- Two attractive charges (2.44 and 2.67 Å) F2- Hydrogen bond	F2- Pi-Pi Stacked (3.97 Å)	Van der Waals	C1- Hydrogen bond (2.21 Å)	N/A	C1- Hydrogen bond (2.27 Å)	Van der Waals	N/A	Van der Waals

			(2.21 and 2.53 Å)	(2.19 Å)								
PR	3537	-7.2	P1- Hydrogen bond (2.48 Å)	P1- Attractive charge (2.26 Å)	Van der Waals	R2- Hydrogen bond (2.21 Å)	P1- Hydrogen bond (2.40 Å)	R2- Hydrogen bond (2.09 Å)	P1- Hydrogen bond (2.27 Å) R2- Hydrogen bond (2.74 Å)	R2- Hydrogen bond (2.46 Å)	N/A	N/A
TF	8185	-7.1	T1- Hydrogen bond (2.44 Å)	T1- Attractive charge (2.11 Å)	Van der Waals	F2- Hydrogen bond (2.12 Å)	T1- Hydrogen bond (2.19 Å)	F2- Hydrogen bond (1.93 Å)	T1- Hydrogen bond (2.25 Å)	F2- Hydrogen bond (2.42 Å)	N/A	Van der Waals
DR	10091	-7.0	D1- Hydrogen bond (2.59 Å)	D1- Two Attractive charges (2.53 Å)	Van der Waals	Van der Waals	D1- Hydrogen bond (2.59 Å)	N/A	D1- Hydrogen bond (2.21 Å)	N/A	N/A	Van der Waals
LR	9213	-6.8	L1- Two Hydrogen bonds (3.01 and 3.08 Å) R2- Hydrogen bond (2.32 Å)	L1- Two Attractive charges (2.16 and 3.07 Å)	Van der Waals	Van der Waals	R2- Pi-Cation (4.91 Å) R2- Hydrogen bond (2.30 Å)	N/A	R2- Hydrogen bond (2.17 Å)	Van der Waals	R2- Two Hydrogen bond (2.24 and 2.46 Å) R2- Attractive charge (4.54 Å)	L1- Metal-Aceptor (2.24 Å)
IL	9079	-6.5	I1- Hydrogen bond (2.22 Å)	I1- Attractive charge (2.74)	L2- Hydrogen bond (2.83 Å)	N/A	I1- Hydrogen bond	N/A	I1- Hydrogen bond (2.41 Å)	N/A	N/A	Van der Waals

				L2- Hydrogen bond (2.37 Å)			(2.10 Å) L2- Pi-Alkyl (5,35 Å)					
AR	7742	-6.4	A1- Two Hydrogen bonds (2.60 and 2.69 Å)	A1- Two Attractive charges (2.63 and 2.65 Å)	Van der Waals	R2- Hydrogen bond (2.10 Å)	A1- Hydrogen bond (2.31 Å)	R2- Hydrogen bond (1.94 Å)	A1- Hydrogen bond (2.18 Å) R2- Carbon Hydrogen bond (2.75 Å)	R2- Hydrog en bond (2.67 Å)	N/A	N/A
DG	7681	-5.8	D1- Hydrogen bond (2.63 Å) G2 - Two Hydrogen bonds (2.33 and 2.70 Å)	D1- Two Attractive charges (2.39 and 2.82) G2 - Hydrogen bonds (2.05 Å)	Van der Waals	N/A	D1 - Hydrogen bonds (2.23 Å)	N/A	D1 - Hydrogen bonds (2.33 Å)	N/A	N/A	Van der Waals
VK	7558	-5.7	K2- Two Hydrogen bonds (2.26 and 2.62 Å)	K2- Attractive charge (2.21 Å)	Van der Waals	V1- Hydrogen bond (2.63 Å)	K2- Hydrogen bond (2.06 Å)	Van der Waals	K2- hydrogen bond (2.26 Å)	V1- Hydrog en bond (2.02 Å)	N/A	Van der Waals

Table S9. ADMET characteristics of ACE-I inhibitory peptides with EC50 <200 µM from chickpea proteins.

**Peptide/ligand**	**Lipinski rules**	**Human intestinal absorption**	**ED**	**Human oral bioavailability 20%**	**ED**	**Human oral bioavailability 30%**	**ED**	**VD (L/kg)**	**ED**	**T 1/2 (h)**	**Rat Oral Acute Toxicity (mg/kg)**	**ED**
Optimal values	MW<=500;<br>logP<=5;<br>Hacc<=10;<br>Hdon<=5	HIA<30%= + ;<br>HIA>30%= -	-	≥20%: F20-;<br><20%: F20+	-	≥30%: F30-;<br><30%: F30+	-	0.04-20	-	0.5	> 500	-
Captopril	Accepted	(--)	🟢	(---)	🟢	(---)	🟢	0.307	🟢	0.86	(---)	🟢
Lisinopril	Accepted	+++	🟠	(--)	🟢	++	🟠	0.494	🟢	0.709	(---)	🟢
VVF	Accepted	(---)	🟢	(---)	🟢	(---)	🟢	0.26	🟢	0.872	-	🟡
VAF	Accepted	(---)	🟢	(---)	🟢	(---)	🟢	0.25	🟢	0.859	(---)	🟢
RY	Accepted	-	🟡	+++	🟠	+++	🟠	0.395	🟢	0.83	(--)	🟢
IVR	Accepted	(---)	🟢	(---)	🟢	(---)	🟢	0.471	🟢	0.514	(--)	🟢
RF	Accepted	(+)	🟡	(-)	🟡	+++	🟠	0.368	🟢	0.824	(--)	🟢
TF	Accepted	(---)	🟢	(---)	🟢	(---)	🟢	0.419	🟢	0.803	(---)	🟢
VF	Accepted	(---)	🟢	(---)	🟢	(---)	🟢	0.254	🟢	0.872	-	🟡
SF	Accepted	(+)	🟡	(---)	🟢	(---)	🟢	0.683	🟢	0.805	(---)	🟢
AF	Accepted	(---)	🟢	(---)	🟢	(---)	🟢	0.249	🟢	0.869	(--)	🟢
YL	Accepted	(---)	🟢	(---)	🟢	(---)	🟢	0.299	🟢	0.909	(--)	🟢
LR	Accepted	(---)	🟢	(---)	🟢	(---)	🟢	0.549	🟢	0.399	(--)	🟢
PR	Accepted	++	🟠	+++	🟠	+++	🟠	0.614	🟢	0.788	(---)	🟢
DR	Accepted	+++	🟠	++	🟠	(-)	🟡	0.819	🟢	0.542	(---)	🟢
CF	Accepted	(--)	🟢	(+)	🟡	(---)	🟢	0.255	🟢	0.901	-	🟡
IL	Accepted	(---)	🟢	(---)	🟢	(---)	🟢	0.39	🟢	0.851	-	🟡
AR	Accepted	(---)	🟢	(---)	🟢	(---)	🟢	0.506	🟢	0.395	(---)	🟢
DG	Accepted	(---)	🟢	(---)	🟢	(---)	🟢	0.274	🟢	0.782	(---)	🟢
VK	Accepted	(--)	🟢	(---)	🟢	(---)	🟢	0.599	🟢	0.732	(---)	🟢
IW	Accepted	(---)	🟢	(---)	🟢	(---)	🟢	0.255	🟢	0.91	(++)	🟠
KF	Accepted	++	🟠	(---)	🟢	(---)	🟢	0.415	🟢	0.835	(--)	🟢

MW: molecular weight; logP: logarithm of octanol-water partition coefficient; ED: Empirical decision; Hacc: hydrogen bond donors; Hdon: hydrogen bond acceptors; VD: Volumen distribution; T 1/2 (h): half-life-time; :Excellent; 🟢 ;Medium; 🟡 ; Poor: 🟠
